# Attachment and coping in psychosis in relation to spiritual figures

**DOI:** 10.1186/s12888-015-0617-4

**Published:** 2015-10-07

**Authors:** Philippe Huguelet, Sylvia Mohr, Isabelle Rieben, Roland Hasler, Nader Perroud, Pierre-Yves Brandt

**Affiliations:** 1 Division of Adult Psychiatry, University Hospital of Geneva and University of Geneva, Rue du 31-Décembre 8, 1207 Geneva, Switzerland; 2 Faculty of Theology, Lausanne University, BFSH 2, 1015 Lausanne, Switzerland; 3 Department of Mental Health and Psychiatry, Division of General Psychiatry, Secteur Eaux-Vives, University Hospital of Geneva, Rue du 31 Décembre 8, 1207 Geneva, Switzerland

**Keywords:** Attachment, Psychosis, Adult Attachment Interview (AAI), Symptoms, Spiritual coping, Schizophrenia

## Abstract

**Background:**

Studies have found higher levels of insecure attachment in individuals with schizophrenia. Attachment theory provides a framework necessary for conceptualizing the development of interpersonal functioning. Some aspects of the attachment of the believer to his/her spiritual figure are similar to those between the child and his/her parents. The *correspondence hypothesis* suggests that early child-parent interactions correspond to a person’s relation to a spiritual figure. The *compensation hypothesis* suggests that an insecure attachment history would lead to a strong religiousness/spirituality as a compensation for the lack of felt security. The aim of this study is to explore attachment models in psychosis vs. healthy controls, the relationships between attachment and psychopathology and the attachment processes related to spiritual figures.

**Methods:**

Attachment models were measured in 30 patients with psychosis and 18 controls with the AAI (Adult Attachment interview) in relationship with psychopathology. Beliefs and practices related to a spiritual figure were investigated by qualitative and quantitative analyses.

**Results:**

Patients with psychosis showed a high prevalence of insecure avoidant attachment. Spiritual entities functioned like attachment figures in two thirds of cases. Interviews revealed the transformation of internal working models within relation to a spiritual figure: a compensation process was found in 7 of the 32 subjects who showed a significant attachment to a spiritual figure.

**Conclusions:**

Attachment theory allows us to highlight one of the underlying dimensions of spiritual coping in patients with psychosis.

## Background

According to the theory of attachment, maintaining the bond to an attachment figure responds to a fundamental need related to survival in mammals. Bowlby [1] distinguished four characteristics of attachment: proximity maintenance (desire to be near the attachment figure), safe haven (returning to the attachment figure for comfort and safety in stressful situations), secure base (the attachment figure acts as a base of security from which the child can explore the environment), separation distress (anxiety that occurs in absence of the attachment figure). According to these criteria, attachment relationship may be secure or insecure [2, 3]. As a result of early experiences relating to primary caregivers, adults who are *securely* attached see themselves as worthy of love, tend to have high self-esteem, and trusting long-term relationships as well as the ability to share feelings with other people. Adults who are *insecurely* attached are more likely to have low self-esteem, to consider themselves as not worthy of love and to doubt the reliability of others or to expect others to be rejecting. *Insecure-avoidant (or dismissing)* attached adults try to regulate their emotions by avoiding feeling the need for closeness or avoiding themes related to separation as well as close relationships. *Insecurely-preoccupied* attached adults constantly and consciously experience fear of being abandoned and are prone to ask others permanently for reassurance [4].

Concerning the intersection between attachment theory and psychopathology, attachment insecurity may *non-specifically* contribute to mental disorders such as mood disorders, personality disorders, and anxiety disorders with several pathways involving effects on self-representation, emotion regulation and problems in interpersonal relations; vice versa, psychological problems could increase attachment insecurity [5].

For the specific case of psychosis, studies using a standardized instrument, such as the AAI (Adult Attachment Interview) [6] found high levels of insecure attachment in individuals with schizophrenia [7–9]. Dozier [7] studied a heterogeneous sample of subjects with psychiatric disorders, among whom only 10 suffered from psychosis. They were more often characterized by an insecure attachment, yet without reaching a significant statistical difference due to this small sample size. Macbeth et al. [9] showed in 34 patients with first psychotic episode that about 3 out of four of them displayed an insecure attachment, most of them of the dismissing category. Gumley et al. [8] showed similar results in 54 patients with first psychotic episode. It should be noted that those authors showed that the narrative method used by the AAI could be used reliably with these psychotic subjects, despite the fact that some of them may feature positive symptoms at the time of assessment.

Overall, non-supportive attachment figures may be understood as a psychosocial risk factor for psychosis, among others [10]. Indeed, taking into account the multi-causal aetiology of schizophrenia, involving both biological factors, e.g. genetic, infectious, obstetrical [11] and psychosocial (e.g. traumatic life events), it is unlikely that a history of non-supportive attachment figures represents by itself an exclusive causal agent.

However, although nonspecific, insecure attachment may lead to specific processes related to the deactivation of positive and negative affects. This may have a role in the unfolding of negative symptoms (see [8]). Also, according to these authors, attachment stability may help subjects to recover from positive symptoms with help of their insight capacities and through a shorter duration of untreated psychosis in the beginning of the disorder.

Overall it is likely that the unavailability of supportive attachment figures may inhibit the development of adaptive cognitive strategies to cope with negative events [9], leading to emotional distress in relation to psychotic symptoms. Conversely, and independently of the quality of attachment brought by caregivers, the process leading ultimately to a full blown psychotic condition may, at least in some cases, involve an impaired premorbid functioning characterized *inter alia* by an avoidance of attachment. This process may ultimately lead to a psychotic syndrome characterized by both negative symptoms and blunted relationships similar to avoidant/dismissing attachment. This latter possibility is in accordance with research showing that patients with thought disorders may appear as dismissing with respect to loss or abuse. Yet that could happen because of their thought disorders rather than as consequence of a failure of caregivers [12].

As stated by Granqvist & Kirpatrick [13], “…the form of “love” experienced in the context of the relationship with God resembles much more closely the prototypical attachment of a child to his or her mother”. Indeed the four above-mentioned criteria of attachment could be found in the relationship of the believer with a spiritual figure. From this perspective, it is suggested that some aspects of the attachment of the believer in relation to his/her spiritual figure are similar to that of the child in relation to his/her parents, that is they serve the function of obtaining/maintaining a sense of felt security when in distress [14]. Two hypotheses have been derived from attachment theory concerning relations between religion and attachment style. The *correspondence hypothesis* suggests that there is a correspondence between early child–parent interactions on the one hand and a person’s ability to cope in relation to a spiritual figure on the other. According to this hypothesis, a secure attachment history would enable a person to use a spiritual figure as an attachment figure, which proximity would help regulate affect. The *compensation hypothesis* suggests that an insecure attachment history would lead to a strong religiousness/spirituality and hence to a possible use of God as a surrogate attachment figure. This mechanism would represent a compensation for the lack of felt security helping to regulate distress [13, 15].

Rationales for our research are based on the fact that studies have found higher levels of insecure attachment in individuals with schizophrenia and moderate associations with greater positive, negative, and affective symptoms [16]. Also patients with schizophrenia often use religious coping, in both positive or negative ways [17], and in particular, religious figures have been found to be involved in the coping of these patients with schizophrenia, as described in general populations [12, 15].

The first aim of this study was to explore attachment models among patients with psychosis in comparison with healthy subjects. Also, we studied the relationship between symptoms and attachment categories and looked at the proportion of trauma reported by subjects, in relation both with psychopathology and attachment categories.

The second goal was to assess whether beliefs or practices related to spiritual figures were associated with attachment processes from the perspective of the correspondence and compensation hypotheses and whether they had an influence on symptoms and coping strategies.

## Methods

### Sample

Participants from the clinical group were stabilized ambulatory patients aged from 18 to 65 followed-up at a clinic of the University Hospital of Geneva who met the ICD-10 [18] diagnostic criteria for schizophrenia or other chronic psychoses. All patients meeting these criteria according to medical records were listed. Participants were randomly selected from this list, without application of any spirituality-related criteria. Fifty-one patients were contacted, seventeen of whom refused to participate because of the length of the research procedure, which meant that 34 patients were recruited. Of these patients, two refused to collaborate with the assessment of attachment models. Two patients withdrew after they had participated in the whole procedure. The Adult Attachment Interview (AAI) [6] could not be coded for two patients, as they did not express themselves enough during the interview, because their childhood memories were emotionally too challenging.

We recruited the non clinical group as comparator for the prevalence of the different styles of attachment. Subjects were contacted by the snow-ball method with the criteria of matching to patients according to age, sex, education level of parents, and as far as possible, to cultural origin. Religious associations of different traditions were contacted. We recruited only religious non clinical subjects for the following reasons: we had planned to recruit patients and for each of them to recruit a healthy subject controlling for age (+/−5 y.o.), gender, parental educational level and religiosity (i.e. having any kind of spiritual or religious beliefs or practices). When processing this recruitment, it appeared that *all patients* were religiously involved, i.e. a trend shown by our previous studies [17]. Hence it was not possible to reach our goal, which was to have a sample of 20 religious patients and 10 non religious patients. In consequence only healthy subjects who were religiously involved have been enrolled. Due to resource limitations for qualitative analyses, twenty potential controls were recruited and screened for Axis I psychiatric diagnoses. Two had to be excluded due to meeting criteria of a psychotic disorder.

### Assessment

The research was approved by the ethics committee of the University Hospital of Geneva and participants gave their written informed consent. Parents’ religious affiliation, practices and beliefs, as well as patients’ own spirituality during their childhood were investigated with a coding grid. Participants were then asked to describe their life experiences including anything they considered to be a change in their spiritual beliefs or practices. Participants’ current spiritual beliefs and practices were investigated, as well as whether these beliefs included a privileged spiritual figure. Then participants’ representations of these spiritual figures and possible internal working models (IWM, i.e. a set of beliefs and expectations about attachment to caregivers) related to them were assessed. Finally, we searched for coping strategies and their efficiency at a qualitative level and on a quantitative self-report scale.

As no validated questionnaire exists in French to assess attachment to spiritual figures among psychotic patients, the AAI [6] (French version by Blaise Pierrehumbert) was used to assess attachment models. The AAI is a semi-structured interview which provides researchers with a standardized method to assess adult mental representations of childhood attachment experiences, such as relation to attachment figures, loss of loved ones and possible traumatic experiences as well as the influence of these experiences as perceived by the participant. It is designed to detect inconsistencies in the participant’s discourse and the latter’s ability to access childhood memories. Inconsistencies between the semantic and episodic memories related to attachment are collected, as they indicate the dissociation of painful affect and highlight specific emotional regulation processes. The central task for participants is that of producing and reflecting on memories related to attachment, while at the very same time maintaining coherent and collaborative discourse with the interviewer [19]. This semi-structured interview involves twenty questions and takes about one hour to administer. Each interview should be assessed for the following categories: Secure/Autonomous, Dismissing, Preoccupied. Both the AAI and the semi-structured interview on spiritual figures were audio-taped and transcribed verbatim. All the interviews were conducted by a well-trained PhD-level psychologist (IR). The AAI responses were coded according to Mary Main’s methodology by a blind certified coder. As the AAI led to a high level of distress for most of our patients, *disorganization*, a category of attachment sometimes considered in the attachment literature, was not systematically explored during the interview according to sensibility to patients’ states.

For assessing attachment to spiritual figures, an analytical grid was constructed based on both the validated spiritual coping grid developed at the University Hospital of Geneva [20] and on the description of IWMs in the literature [1, 21–24]. This semi-directive interview (original version in [25]) examines the contents of religious and spiritual beliefs and practices, the importance of such beliefs and practices in the life of the person, how they are employed in attempts to confront difficulties and the impact that they have on well-being. This interview checks also the presence of religious education during childhood, as well as any subsequent religious changes considered as significant by the person. More specifically, questions about the participants' relations to principal attachment figures were asked with respect to spiritual figures. Questions were posed in such a way as to invite a general discussion of oneself as well as encouraging a narration about very specific situations and conditions.

Childhood traumatic experiences in relation to attachment figures were extracted from the AAI with a focus on the following points: Multiple traumatic experiences, Separation from first attachment figure, Repeated separation from first attachment figure, Sexual abuse, Violence from parents, Parental psychiatric Disorder traumatic for the subject.

For patients, the Brief Psychiatric Rating Scale (BPRS) [26] was used to evaluate current symptoms. This evaluation consists of 24 dimensions. Items 1 to 14 are evaluated on the basis of the answers given by the patient, items 15 to 24 on the basis of patient's behavior that can be observed.

Diagnosis were estimated by comparisons of data from medical chart, current patient’s psychiatrist, and by the Structured Clinical Interview for DSM-IV Axis I Disorders (SCID) [27]. SCID-NP (a simplified version permitting one to identify the presence of an Axis I symptom in a “Non-Patient” sample [28]) was used to discriminate the actual presence of any psychiatric diagnosis in the control group.

### Data analysis

Links between participants’ socio-demographical data, patient’s age at first episode, the intensity of the symptoms for patients as well as all the material brought out by qualitative analysis (attachment models, spiritual beliefs and practices relating to a spiritual figure and spiritual coping strategies) were analyzed statistically using the Chi-square and independent T-test with a probability level of < .05.

## Results

### Attachment across groups

Socio-demographical and clinical variables are presented in Table 1. There was no difference on socio-demographic and clinical variables when comparing the six patients who dropped-out from the study from the ones who stayed in.

**Table 1:** Demographic and clinical characteristics

		Patients (n = 28)		Controls (n = 18)	
		Mean (sd)		Mean (sd)	
Age		41.6 (10.05)		41.3 (12.01)	
		n	%	n	%
Gender	Male	20	71	11	61
	Female	8	29	7	39
Marital Status	Single	22	79	6	33
	Married	2	7	11	61
	Divorced or separated	4	14	1	6
With children		4	14	8	44
Living	In sheltered home	13	46		
	Alone	8	29	4	22
	In couple/With family	2	7	14	78
	At parent’s place	5	18		
Diagnosis	Paranoid-schizophrenia	22	79		
	Schizoaffective disorder	6	21		
Age at onset (mean, sd)		25.43 (8)			
	Before 26 years	20	71		
	After 32 years	8	29		

The prevalence of attachment is described in Table 2, where data from the meta-analysis of Bakermans-Kranenburg and van Ijzendoorn [29] was entered. Patients displayed same prevalence of secure attachment as the clinical population (χ2(1) = 1.68, *p* = .19); and less than our control group (χ2(1) = 11.68, *p* < .01), as well as the non-clinical population (χ2(1) = 14.34, *p* < .01).

**Table 2:** Attachment distribution between groups

	Secure		Insecure dismissing		Insecure preoccupied		Total
	n	%	n	%	n	%	n
Patients*	6	21	19	68	3	11	28
Control	13	72	3	17	2	11	18
Clinical Population**	33		39		28		3389
Non-clinical Population***	57		29		14		4392

Clinical and non-clinical populations (Bakermans-Kranenburg, M. J., van IJzendoorn, M. H., [43])

*Patients Vs. control group: χ2(1) = 11.68, *p* < .01

**Patients Vs. clinical population: χ2(1) = 1.68, *p* = .19

***Patients Vs non-clinical population: χ2(1) = 14.34, *p* < .01

According to the BPRS, total score was significantly lower in patients with a secure attachment model compared to those with other attachment styles (see Table 3); specifically for anxiety (m = 2.33+/−.82 Vs. m = 4.23+/−1.02, T = 4.18, *p* < 0.001), depression (m = 2.33+/−1.21 Vs. m = 3.86+/−1.08, T = 3.00, *p* < 0.01), suspiciousness (m = 1.50+/−.84 Vs. m = 3.50+/−1.10, T = 4.11, *p* < 0.001), unusual thought content (m = 1.83+/−2.04 Vs. m = 3.50+/−1.44, T = 2.30, *p* < 0.05), and motor retardation (m = 1.00+/−.00 Vs. m = 1.95+/−1.05, T = 2.21, *p* < 0.05). In addition, insecure attachment was associated with an earlier onset of psychosis (82 % before 26 years-old vs. 50 % for late onset, χ2(1) = 5.43, *p* = < .02).

**Table 3:** Attachment and symptoms

	Secure		Insecure (Dismissing and preoccupied)		Total		Wilcoxon rank test	
	n = 6		n = 22		n = 28			
BPRS^a^	Mean	s.d.	Mean	s.d.	Mean	s.d.	W	p.
Total Score	39	10	54	7	51	10	35	.01

^a^Brief Psychiatric Rating Scale

Table 4 shows data on categories of trauma and attachment. A high prevalence of child trauma related to attachment figures was found in the psychiatric sample when compared to our control sample. Twenty-one patients out of 28 and 8 controls out of 18 experienced traumatic childhood experiences related to their attachment figures (χ2(1) = 4.39, *p* < .04). For the whole sample only, insecure attachment was associated with separation from first figure of attachment (33 % for insecure vs. 6 % for secure, χ2(1) = 5.16, *p* < .02), and multiple traumatic experiences (48 % for insecure vs. 16 % for secure, χ2(1) = 5.15, *p* < .02). Due to the small sample size, we were not able to find a significant difference in terms of percentage of subjects with trauma between the patients groups and the controls.

**Table 4:** Traumatic childhood experiences related to attachment figures and categories of attachment between groups

	Patients			Controls			Total		
	n	S^ecure^	I^nsecure^	n	S^ecure^	I^nsecure^	n	S^ecure^	I^nsecure^
	28	6	22	18	13	5	46	19	27
Traumatic experience*	21	3	18	8	6	2	29	9	20
Multiple traumatic experiences	12	1	11	4	2	2	16	3	13
Separation from first attachment figure	9	1	8	1	0	1	10	1	9
Repeated separation from first attachment figure	5	1	4	4	2	2	9	3	6
Sexual abuse	5	0	5	1	1	0	6	1	5
Violence from parents	11	2	9	2	1	1	13	3	10
Parental psychiatric									
Disorder traumatic for the subject	5	0	5	1	1	0	6	1	5

*Patients Vs. Controls, χ2(1) = 4.39, *p* < .04

### Attachment and coping with spiritual figures

Sixty-four percent of patients and 78 % of controls (i.e. 32/46 participants overall) believed in a spiritual figure who functioned like an attachment figure for them, according to our qualitative coding grid. According to the four criteria defining a secure attachment figure, 29 % of patients had a secure attachment to their spiritual figure, whereas 36 % had an insecure one. Among the controls, 67 % had a secure attachment and 11 % had an insecure attachment to their spiritual figure. Here are some of patients’ excerpts illustrating secure and insecure attachment to a spiritual figure (Tables 5, 6, 7, 8). It should be noted that coding was not based on these excerpts alone but on the content and coherence of the entire interview.

**Table 5:** Attachment behavior: looking for proximity

Secure	Insecure
Patient 11	Patient 22
*« Sometimes when I’m walking in the street, when I’m in my bed, in fact anywhere, at any time, I can talk or whisper to God »*	*« God is everywhere, but I don’t want to be close to him, I just want him to help me out »*

**Table 6:** Safe haven in times of distress

Secure	Insecure
Patient 13	Patient 15
*« When I feel a little sad, then I pray to the Lord, sometimes I read the Bible and I feel better afterwards »*	*« I’m scared he’ll get tired of me if I ask for help too often »*

**Table 7:** Secure base

Secure	Insecure
Patient 14	Patient 20
*« It gives you a feeling of security, it’s like always having a protecting angel who watches your steps »*	*« In my everyday life I would say it gives me a mini protection, but nothing I can count on »*

**Table 8:** Separation distress

Secure	Insecure
Patient 5	Patient 19
*« I could not live without him anymore, I don’t know how others can! »*	*« Oh, I wouldn’t care if he wasn’t there anymore, it’s an illusion anyway »*

### Coping with spiritual figures and other characteristics

Characteristics of subjects, according to attachment towards a spiritual figure, are important to consider. Indeed some symptoms (e.g. delusions with religious content) may alter God’s image as an attachment figure; conversely, having a positive religious coping involving a secure attachment to a spiritual figure may have a soothing effect, hence possibly improving some symptoms such as anxiety, lowered mood and even some psychotic features [17].

Concerning the relationship between attachment and symptoms, it appeared that patients who featured a *secure* attachment to primary care giver and/or to a spiritual figure had a better symptom profile for somatic concern (Secure N = 11 Vs. Unsecure N = 17: m = 1.73+/−.90 Vs. m = 2.94+/−1.56, T = 2.33, *p* < 0.05), anxiety (m = 2.73+/−.90 Vs. m = 4.53+/−.87, T = 5.26, *p* < 0.001), depression (m = 2.82+/−1.25 Vs. m = 4.00+/−1.06, T = 2.69, *p* < 0.01), guilt (m = 1.55+/−.52 Vs. m = 2.88+/−1.62, T = 2.64, *p* < 0.05), suspiciousness (m = 2.00+/−1.18 Vs. m = 3.76+/−.90, T = 4.47, *p* < 0.001), conceptual disorganization (m = 1.27+/−.47 Vs. m = 2.47+/−1.42, T = 2.69, *p* < 0.05), emotional withdrawal (m = 1.55+/−.82 Vs. m = 2.59+/−1.33, T = 2.33, *p* < 0.05) and motor retardation (m = 1.09+/−.30 Vs. m = 2.18+/−1.07, T = 3.25, *p* < 0.001). However, beyond this quite large and heterogeneous symptom profile for this subgroup, hallucinations profile was quite similar across subgroups (m = 2.91+/−2.12 Vs. m = 2.88+/−1.41, T = 0.04, *p* < 0.97).

For other characteristics, having a secure attachment towards a spiritual figure was positively linked with increased self-esteem and self-reports related to spiritual coping in both patients and controls (χ2(1) = 17.06, *p* = < 0.001). Also participants who invested their spiritual figure with the qualities of a secure attachment figure were more likely to report that these spiritual beliefs helped them to trust others (χ2(1) = 10.25, *p* < 0.01) and that they were able to find comfort in their relation to their spiritual figure (χ2(1) = 4.66, *p* < 0.05). Finally, participants with a secure attachment towards a spiritual figure reported a better ability to deal with symptoms like depression (χ2(1) = 10.13, *p* < 0.01) and anxiety (χ2(1) = 6.34, *p* < 0.05).

### Relation between IWM for primary caregivers and IWM for spiritual figures

Among the 32 participants for whom the spiritual figure worked as an attachment figure, 22 (12 patients and 10 controls) projected the same attachment model onto their spiritual figure as the one developed in their relation with their parents. Three participants (one patient and two controls) showed a secure attachment towards their primary caregiver and developed an insecure attachment to their spiritual figure, whereas seven participants (five patients and two controls) with insecure attachment to primary caregivers had a secure attachment towards a spiritual figure. The five patients experienced fewer symptoms such as anxiety (T(22) = 3.01, *p* < 0.01) and suspiciousness (T(22) = 2.27, *p* < 0.05) compared with the 17 patients who had no secure attachment either to primary caregivers or to spiritual attachment figures.

All patients for whom we identified a compensation process mentioned having a significant emotional experience which led them to trust their spiritual figure. Here are two examples of patients’ reports:

#### Patient 5

*Then I told him: Lord I hate you with all my heart, with all my soul, with all my spirit, with all my strength! The following second I heard words that felt like an enlightenment: « Because to me, you can say all you feel: I’m love, I’m stable, I’m based on freedom. Our relationship started then and today I trust him with everything! God has opened me up to something really important. I’m allowed to be angry with him, to go crazy sometimes, even to yell at him, but he will never take his love from me. He showed it several times to me and we have conversations together, I hear him out loud »*

#### Control 19

« *At that time I did something that I was not supposed to do, I was scared to lose Allah’s love, but to my great surprise, I could feel that he was still there, stronger than ever*»…« *Since that experience, I have had a different relation with him, I feel it’s even purer, it’s even more powerful*».

## Discussion

In this research we found that patients with chronic psychosis featured a higher prevalence of insecure attachment. The symptomatology of patients with insecure attachment was more severe. Also, a high prevalence of childhood trauma related to attachment figures was observed in patients with psychosis compared to our control sample. A large majority of subjects in both groups believed in a spiritual figure that functioned like an attachment figure. Amongst them, a compensation process was observed in some subjects, i.e. they showed a stable attachment to a spiritual figure in the context of a primary insecure attachment towards caregivers.

### Insecure attachment style and psychosis

The first aim of this study was to explore attachment models among patients with psychosis. They featured a high prevalence of insecure attachment (mostly dismissing/avoidant) as compared with the control group. The results for the latter group can be compared with those of the meta-analysis of van IJzendoorn and Bakermans-Kranenburg [29] on a group of 4392 non-clinical subjects showing a 57 % rate of secure attachment. Indeed most studies to now have shown a higher prevalence of insecure attachment (mostly dismissing/avoidant) in patients with psychosis [7, 9, 30–37] as compared with non-clinical samples [29].

Insecure dismissing attachment displays strong avoidant cognitive, emotional and behavioral attitudes intended to avoid activating the attachment system. The discrepancy between the episodic and the semantic memory highlighted by the AAI illustrates this specific psychological mechanism accurately. When an emotionally challenging life event related to an attachment figure enhances suffering, episodic memory tends to dysfunction (often in an adaptive way), hence allowing subjects to construct a corrected representation of their primary attachment relations and maintain the bond. Benedetti [38] as well as others emphasized the presence of dysfunctional ways of communication in the families of psychotic patients. This is often related to experiences of intrusion, rejection and extreme invalidation of children’s feelings in the context of their relation to a primary caregiver. In such a context, where the deep cognitive and emotional distortions required are useful to the child in maintaining the bond with his/her attachment figures, it seems logical to consider that, when growing up, individuals could be prone to develop an affect regulation mechanism that may contribute at some point to a rupture with socially shared reality. Therefore, repetitive experiences of intrusion, rejection and invalidation of one’s feelings in association with genetic vulnerability might significantly increase the risk of psychosis as demonstrated in epidemiological surveys (e.g. [39]). This may be reflected in more recent work such as the one conducted on mentalization [40], which relates historically but also conceptually to the concept of attachment. The capacity to mentalize, defined as “… the activity of understanding behavior in relation to mental states such as thoughts and feelings” [41], develops optimally in the context of a relationship with a stable attachment. Lack of mentalizing is found in various psychiatric disorders such as autism (in this case probably related to a neurobiological disorder), borderline personality disorder and schizophrenia [42]. Therefore a history of an unstable relationship with caregivers may lead to insecure attachment and later on to altered mentalizing capacities, i.e. an important limitation on the lives of people suffering from psychosis. In this perspective, MacBeth et al*.* [9] showed that, independently from symptomatology, reflective function (RF, a measure of mentalization) was higher in psychotic patients with secure attachment than in those with insecure attachment.

As mentioned above, we cannot state that an experience of insecure attachment may represent per se a *specific* causal factor for psychosis. First, the prevalence of insecure attachment in psychosis was the same as that reported in other clinical populations [43]. Second, there are patients with psychosis who do not report a history of unreliable attachment. Third, some patients, both in MacBeth et al. [9] study, Gumley et al. [8] study, and in our data, feature a current secure attachment. Fourth, research shows that a history of unstable relationships may lead to various psychiatric conditions, depending on other factors in the array of biology, psychology or social context. Therefore, unreliable attachment affects the illness trajectory and process and is not a cause of the illness.

### Attachment and symptoms

In the two researches conducted on first-episode patients in Scottland [8, 9] no association was identified between attachment categories and symptoms in psychosis, whereas, in our study, secure attachment was associated with less positive symptoms, anxiety/depression, and agitation/mania. This discrepancy could be explained by the nature of the samples (first-episode patients vs. patients with chronic psychosis). Indeed, the process of dealing with psychosis involves cognitive and emotional schemas which may become more visible over time. Therefore, an association between symptoms and a coping process could be more evident in chronic patients rather than first-episode patients.

Some studies code the AAI along two dimensions by the Q-sort method; i.e. security/anxiety and repression/preoccupation. With this procedure, no associations were found between baseline clinical severity and attachment for 31 patients at-risk of psychosis [36]; whereas, for 40 chronic patients, insecure attachment was associated with more positive symptoms, especially delusion, hallucination and suspiciousness [44]. Data from several studies using auto-questionnaires to assess attachment dimensions provide evidence for a link between avoidant attachment and positive and negative symptoms [16].

The association of insecure attachment with positive symptoms warrants some comments, beyond the fact that persistent positive symptoms may *per se* lead to some “insecure attachment”, or conversely that insecure attachment would lead to social or cognitive problems leading to positive symptoms, in the context of an altered process of affect regulation [16]. In the field of diabetes, Ciechanowski et al. [45] showed that dismissing attachment in the setting of poor patient-provider communication was associated with poorer treatment adherence in patients with diabetes. The differences between groups found by these authors, in medication adherence and glucose monitoring suggested a disengagement from treatment by patients who exhibit dismissing attachment, particularly in the absence of good patient-provider communication. Translated into the field of psychosis treatment, both in its pharmacological and psychosocial dimensions, this work may suggest some hypotheses. Indeed, in addition to other features related to their symptoms, patients with psychosis featuring insecure attachment would have more problems with compliance, due to a poor quality of patient-provider relationship. Hence, a worst profile for positive symptoms may be due to a poorer adherence to treatment, amongst other causes. This hypothesis is in line with research showing that insecure attachment with mental health services may alter the course of patients with psychosis [46]. These authors conclude an interesting clinical implication: security of attachment should be assessed in order to identify patients who might experience difficulties in engaging with services and who may need increased input on this issue.

To note, we did not find an association between negative symptoms and attachment categories. That may be related to a “floor effect”, i.e. to the fact that patients included in the research were stabilized. That means that they were supposed to feature no or little positive symptoms, depending on the magnitude of the effect of their neuroleptic treatment. In association, negative symptoms may appear quite stable in the lower range hence not allowing a clear distinction between categories of subjects for this parameter (our data show almost same results for all groups, with a quite small DS). An alternative explanation would be that attachment style may not influence negative symptoms *per se*. However, this hypothesis would be in contradiction with literature showing an association between attachment categories and negative symptoms (see Gumley’s et al. review [16]). Indeed, it is unlikely that the process leading for example to dismissing/avoidant attachment would not entail some behaviors and emotional features which would not be part of negative symptoms. Hence insecure attachment may be related to the deactivation of positive and negative affect [8]. Also, it is unlikely that “primary” negative symptoms would not affect the relationships hence altering the perception of attachment in its assessment.

### Attachment and trauma

Concerning the association between psychosis and a high prevalence of childhood trauma related to attachment figures, the small number of subjects in our research hinder us from drawing conclusions, beyond the fact that our results illustrate (rather than demonstrate) the fact that many patients suffering from psychosis report a history of trauma such as abuse, parental neglect and others. This clinical observation is confirmed by recent epidemiological surveys such as the one of Read et al. [39], or Shevlin et al. [10], the latter showing an additive effect of multiple traumatic experiences. Indeed, in this research, whereas a single trauma type did not appear to increase the risk of psychosis, experiencing two or more types of trauma significantly increased this risk, with dramatic increases associated with experiencing all types of trauma. Picken et al. [47] showed in 110 patients with psychosis and substance misuse, that anxious attachment was associated with a number of interpersonal traumas and post traumatic symptoms, like in other clinical populations.

The issue of the influence of trauma on attachment style in this particular population of patients with psychosis was described in the recent meta-analysis of Gumley et al. [16]. Berry et al. [48] found higher levels of attachment anxiety in subjects who had experienced trauma from caregivers during childhood. Picken et al. [47] found that attachment anxiety was associated with the total number of traumatic events. Keeping in mind our small sample size for this kind of statistics, we note that this literature is in accordance with our results showing for the whole sample an association between insecure attachment and separation from first figure of attachment and multiple traumatic experiences.

### Attachment: clinical implications

The fact that some patients with psychosis feature a secure attachment or an insecure yet preoccupied attachment is another important issue arising from these data. Actually about one out of three patients falls into these two categories. This means that, beyond the stigmatizing view that patients with psychosis may feature an “autistic” relationship (see Laing [49], or classical literature on first rank symptoms, e.g. Andreasen and Black [50]), a third of them may be able to have significant and rewarding relationships (through a secure attachment) or a significant investment to others (although to some extent problematic, i.e. in the cases of preoccupied attachment). This may represent for these patients an important goal in their lives, which should be kept in mind by clinicians who often deny them the actual possibility to build high quality relationship such as friendship or romantic bounds [51]. Indeed, this involves that clinicians should encourage patients to build relationships far beyond the social skills training which is offered in many places (e.g. [52]). That may involve specific psychotherapeutical intervention. In this perspective, targeting mentalization, known to be altered in this population [9, 40] may improve to some extent cognitive, emotional and behavioral attitudes related to attachment. In research on the particular realm of patient-therapist relationship, AAI classification has been shown to predict the kind of collaboration involved in the treatment process [53]. These authors showed that secure and preoccupied patients were more likely, as compared to dismissing patients, to seek emotional closeness with the therapist, dismissing patients were more likely to avoid proximity, and preoccupied patients were more likely to resist the therapist’s support or connection. Even this study was done with patients likely to suffer from disorders other than psychosis, there remains to be assessed whether or not patients with psychosis may feature such pattern. At least this should be considered when treating this particular population of patients with psychosis, taking into account the fact that the quality of social relationships impacts on the disorder’s outcome [54]. In this perspective, Korver-Nieberg et al. [42] associated the category of attachment to the recovery style and suggested the need to improve attachment security in a context of therapeutic relationship, before encouraging people to explore their experiences of psychosis.

### Attachment and spiritual figures

The use of a semi-structured interview and a qualitative coding grid based on attachment theory as well as on the spiritual coping grid developed previously in our research group [17] allowed us to investigate more deeply the process of spiritual coping in relation to spiritual figures. Firstly, we showed that in the great majority of cases spiritual figures functioned like attachment figures. Having a secure attachment to a spiritual figure (even when there was no secure attachment towards primary caregivers) was associated with lower levels of symptoms such as suspiciousness and anxiety and better coping strategy with regard to self-esteem, depression, hope, relation to others and giving a meaning to life. Hence, some aspects of religion may promote “earned security”, because God is perceived as a loving attachment figure [13]. Furthermore, our results showed that even for the 12 participants (10 patients and two controls) for whom the spiritual figure worked like an insecure attachment figure, this characteristic appeared to be associated with a better symptomatic and/or coping profile. Having an insecure spiritual attachment figure still appears to be more useful than having none.

Even if this study, due to its cross-sectional design, is not designed to address causality, in terms of attachment to religious figure’s style vs. level of symptoms, this issue needs to be discussed. Indeed this question is important in two ways. First, one may question whether stable attachment to a spiritual figure may improve symptoms or if conversely a better symptoms profile may allow patients to “build” a stable attachment to a religious figure. Our results show indeed such an association, although for 8 symptoms quite heterogeneous in their nature. Some of those symptoms, mostly in the “emotional” field, may give support to the first formulation (e.g. a “stable attachment to God” may relieve anxiety and depression); conversely some symptoms, more in the “cognitive” domain such as guilt, suspiciousness and conceptual disorganization, may hinder building such a favorable relation with a spiritual figure.

Also it is worth discussing the fact that the presence of psychotic symptoms such as delusions with religious content may alter the way patients report their attachment to spiritual figures. Indeed some studies [55] showed that 15 % of stabilized patients with psychosis feature delusions with religious content. Amongst those, some felt having a relationship with God involving some influence of Him, in a delusional way (e.g. being controlled or even persecuted by Him). In the present study, this kind of phenomenon has been considered as a negative form of coping and the sign of an insecure attachment to the religious figure. Yet we should keep in mind that this may be state dependent, hence a reversible condition possibly related to an increase of positive symptoms. Only repeated assessments may possibly address this issue.

The correspondence and compensation hypothesis could explain some aspects of religiosity from a developmental perspective: the “relational part” of religiosity could be either 1) the result of a secure attachment to a spiritual figure, based on the previous development of a secure attachment towards parents or 2) a secure attachment toward a spiritual attachment figure based on a compensation process following a primary insecure attachment towards caregivers. Such an arrangement was described by Granqvist & Hagekull [15] who reported that parental insensitivity predicted increased importance of the relationship with God.

Attachment models are described as generally stable over lifespan [21]. However, attachment theorists consider that, at some point during lifespan, it is possible to experience different kinds of relationships, these other relations sometimes taking the form of emotionally significant experiences able to transform these IWMs [56]. Therefore, these authors emphasized the need to think in terms of a hierarchy of IWMs over lifespan. This is consistent with our finding that correspondence is more prevalent than transformation in IWMs. The transformations we observed in participants’ IWM in their relation to spiritual figures could be the result of two different processes. Firstly, the relationship could be enhanced by an emotionally significant change experienced by subjects in relation to their beliefs concerning their current relation to fantasized spiritual figure. Secondly, it could be the result of an emotionally significant change that subjects experienced with another relation such as a friend, which was then projected on the representation of the spiritual figure. Our data do not allow us to find out which of these processes is involved. However, Bowlby [23] insisted that only sensitive experiences were able to change IWMs. From this perspective, our qualitative data indicated that all seven participants who modified their IWM in relation to their spiritual figure reported that they had felt sensitive experiences in their bodies and had experienced specific events which were interpreted as the manifestation of a spiritual figure. Hence, patients’ spiritual interpretations of sensitive experiences may represent what Bowlby described as sensitive experiences in the relation to an attachment figure.

### Limits

The strength of our research is definitely the use of clinically powerful instruments (such as the AAI, the semi-structured interview for the assessment of spirituality and the SCID for the confirmation of the diagnosis). The length of the investigation process as well as the sensitivity of the AAI led to some drop outs however. Therefore the small size of our sample definitely represents a limit to the external validity of this research. Some limits are based on conceptual issues. Firstly, as discussed above, the fact that psychosis is associated with a higher rate of insecure avoidant attachment is not per se the proof of a *causality* of attachment disruptions on the later onset of psychosis. This issue pervades the literature on attachment and adult psychopathology (e.g. Dozier et al. [12]). Only prospective studies could provide support for this connection. Until then, it cannot be excluded that insecure avoidant attachment could be one feature of a severe form of psychosis. The same kind of issue arises when considering the correspondence and the compensation hypotheses. We cannot exclude the possibility that those who are able to compensate are also able to develop a global improvement in their ability to cope with interpersonal issues, because of a milder form of psychotic disorder. This could explain why they feature less persistent symptoms.

Finally the fact that patients were to some extend stabilized may not bring about a sufficient symptom range allowing fully efficient statistical analyses: overall 19 patients (68 %) featured at least one moderate or severe psychotic positive symptom in BPRS.

## Conclusions

During the last decades, research on the etiology of psychosis has predominantly focused on various biological factors. The high level of childhood trauma and insecure avoidant attachment in patients with chronic psychosis as well as their association with symptoms and coping abilities lend support to the investigation of psychological and environmental variables in future research on this condition. Among other, the relationship, in particular in terms of causality, between symptoms and attachment categories represents a major issue, due to the obvious clinical implications it entails. Indeed, the present cross sectional research, but also other research involving follow-up [8] cannot disentangle this problem of causality. This warrants further research on the pathways leading both to psychosis and adult attachment insecurity. That should foster clinicians to shift from a fixed, fatalist stance, for example when facing negative symptoms in the field of relationships with significant others, to a view more concerned with an attachment related stance. That would allow clinicians to place attachment in the core of their psychosocial intervention, instead of reducing patients experience to fixed, biological impairments. Another issue important for clinicians is that patients with unstable attachment may need increased attention and specific interventions in current rehabilitation settings. Approaches involving this domain (e.g. those fostering mentalizing capacities) may be useful in this perspective.

Other studies showed that patients with chronic psychosis can use spirituality as a positive resource for coping (e.g. [17]). In this research, one of the underlying dimensions of the attachment theory which supports these previous data has been highlighted. In particular, it appears that some processes involved in spiritual coping might induce deep psychological changes. At the same time, spiritual beliefs and practices may constitute a privileged window through which to examine IWMs and their transformations.
